# Recent Advances in Nanotechnology for the Management of *Klebsiella pneumoniae*–Related Infections

**DOI:** 10.3390/bios12121155

**Published:** 2022-12-10

**Authors:** Mahmood Barani, Hadis Fathizadeh, Hassan Arkaban, Davood Kalantar-Neyestanaki, Majid Reza Akbarizadeh, Abduladheem Turki Jalil, Reza Akhavan-Sigari

**Affiliations:** 1 Student Research Committee, Kerman University of Medical Sciences, Kerman 7616913555, Iran; 2 Medical Mycology and Bacteriology Research Center, Kerman University of Medical Sciences, Kerman 7616913555, Iran; 3 Department of Laboratory Sciences, Sirjan School of Medical Sciences, Sirjan 7616916338, Iran; 4 Department of Chemistry, University of Isfahan, Isfahan 8174673441, Iran; 5 Department of Medical Microbiology (Bacteriology and Virology), Afzalipour Faculty of Medicine, Kerman University of Medical Sciences, Kerman 7616913555, Iran; 6 Department of Pediatric, Amir Al Momenin Hospital, Zabol University of Medical Sciences, Zabol 9861663335, Iran; 7 Medical Laboratories Techniques Department, Al-Mustaqbal University College, Babylon, Hilla 51001, Iraq; 8 Department of Neurosurgery, University Medical Center Tuebingen, 72076 Tuebingen, Germany; 9 Department of Health Care Management and Clinical Research, Collegium Humanum Warsaw Management University, 00014 Warsaw, Poland

**Keywords:** nanotechnology, *Klebsiella pneumoniae*, metallic nanoparticles, antibacterial, detection

## Abstract

*Klebsiella pneumoniae* is an important human pathogen that causes diseases such as urinary tract infections, pneumonia, bloodstream infections, bacteremia, and sepsis. The rise of multidrug-resistant strains has severely limited the available treatments for *K. pneumoniae* infections. On the other hand, *K. pneumoniae* activity (and related infections) urgently requires improved management strategies. A growing number of medical applications are using nanotechnology, which uses materials with atomic or molecular dimensions, to diagnose, eliminate, or reduce the activity of different infections. In this review, we start with the traditional treatment and detection method for *K. pneumoniae* and then concentrate on selected studies (2015–2022) that investigated the application of nanoparticles separately and in combination with other techniques against *K. pneumoniae*.

## 1. Introductions

Infectious diseases such as pneumonia, diarrhea, tuberculosis, and malaria, are leading causes of death, responsible for 2.2 million, 1.8 million, 1.5 million, and 1.2 million deaths, respectively [1]. More than 95% of the deaths happen in low-income and middle-income countries [2]. The notable increase in drug resistance of infectious agents inhibits efficient therapy for diseases [3]. *Klebsiella* species are Gram-negative, nonmotile, encapsulated bacteria found in nature, including surface waters, soil, animals, medical devices, and healthcare environments [4]. They were first identified in the late nineteenth century and became known as Friedlander’s bacterium [5]. *K. pneumoniae* virulence factors play various roles in different *K. pneumoniae* infections. These virulence agents include fimbriae, siderophores, lipopolysaccharide (LPS), porins, outer membrane proteins (OMPs), iron transport systems, efflux pumps, and genes related to allantoin metabolism [6]. This bacterium can produce biofilms on different levels in the body to escape the host defense. In addition, due to the presence of a polysaccharide capsule that acts as an outer shell, it is significantly protected from phagocytosis by polymorphonuclear granulocytes [7]. In general, *K. pneumoniae* has long been known as an organism that causes serious primary infections in immunocompromised people. Still, the number of individuals at risk has increased due to the emergence of excessive virulence strains. Even healthy people and persons with adequate immunity are at risk of becoming infected with the bacterium [8]. *Klebsiella pneumoniae* as an opportunistic pathogen can be easily colonized in human mucous membranes such as the oropharynx and gastrointestinal tract; this colonization appears to be benign [4]. Nevertheless, if this bacterium spreads from the mucosa to other tissues in the body, it can lead to serious and threatening infections such as pneumonia, sepsis, and urinary tract infections (UTIs) [9]. Klebsiella infections cause serious problems in immunocompromised patients, infants, and the elderly. This organism is also one of the bacteria that cause nosocomial infections and community-acquired infections [10]. *K. pneumoniae* can be divided into hospital-acquired pneumonias (HAPs) and community-acquired pneumonias (CAPs). HAPs are much more common than CAPs, and the underlying cause of 11.8% of HAPs is *K. pneumoniae* [11]. One of the most serious and dangerous consequences of *K. pneumoniae* pneumonia and urinary tract infections is bacteremia caused by bacteria entering the bloodstream. *K. pneumoniae* is the second most deadly bacteria among Gram-negative bacteria as a cause of both community- and hospital-based bacteremia [12]. A *K. pneumoniae* pathotype known as hypervirulent *K. pneumoniae* (hvKp) is spreading worldwide. Unlike infections caused by the classical *K. pneumoniae* (cKp), hvKp causes invasive tissue infections in healthy people in the community and often involves multiple sites [13]. In recent years, the resistance to a wide range of antibiotics of *K. pneumoniae* has increased significantly. As a result of this increase in antibiotic resistance, infections such as pneumonia and bacteremia are increasingly threatening human health. Simple infections such as urinary tract infections often do not respond well to treatment [14,15]. The emergence of multidrug-resistant (MDR) bacterial strains is a major threat to patients because, with the spread of this type of antibiotic resistance, most existing treatments fail [16,17]. Several mechanisms of resistance to antibiotic agents, including the product of ion extended-spectrum β-lactamases (ESBLs) and carbapenemases such as *Klebsiella pneumoniae* carbapenemases (KPC), over-activation of efflux pumps, and porin modification, have been reported in *K. pneumoniae*. This is important as the bacterium becomes resistant to all β-lactams, including carbapenems [18]. The emergence of KPC-producing strains has become a significant medical problem. β-lactamases can hydrolyze carbapenem and make this bacterium resistant to a wide range of β-lactam antibiotics. Therefore, treating infections caused by this pathogen has become a considerable challenge [19]. KPCs are frequent in *K. pneumoniae* but may also be seen in other Enterobacteriaceae types, including *Salmonella enterica*, *Enterobacter* species, *Escherichia coli*, *Citrobacter freundii*, and *Proteus mirabilis* [20,21]. Because of the presence of KPC genes, for example, *bla_KPC-2_*, on the plasmid, has a high ability to spread [20,22]. Due to the lack of appropriate alternative therapies, *K. pneumoniae* infections created by ESBL-producing strains and carbapenem-resistant strains have a higher mortality rate than non-resistant bacteria [23]. The emergence of MDR *K. pneumoniae* strains, including colistin-resistant strains, has caused great concern. This type of resistance occurs due to mutations in the *mgrB* gene, which is stably conserved in *Klebsiella* populations and by the plasmids carrying *mcr-1* and *mcr-2* genes [24]. Furthermore, an extensive drug-resistant (XDR) cKp strain, which obtained a segment of a virulence plasmid carrying hvKp, led to a lethal nosocomial outbreak [25]. Today, the search for alternative therapies for antibiotic-resistant *K. pneumoniae* and other bacteria is vital and essential worldwide [26].

Due to rising antibiotic resistance, *K. pneumoniae* infection has become a significant health hazard, restricting effective treatments. Therapies reprogramming lung defenses and improving immune response to clear bacteria may help prevent *K. pneumoniae* infection [27]. Accordingly, in recent years, there has been increased demand for new strategies, pharmaceuticals, and devices to diagnose and treat diseases precisely and competently [28,29]. Many types of nanoparticles, such as graphene, polymers, vesicles, and green synthesized NPs, have been developed as drug delivery systems in cancer and infectious diseases [30,31,32]. The ability of nanomaterial-based therapeutics and diagnostics to overcome established processes linked to acquired drug resistance makes them intriguing tools for treating difficult-to-treat bacterial infections. Additionally, nanoparticles’ distinctive size and physical characteristics enable them to target biofilms and treat resistant illnesses [33]. Many kinds of nanomaterials have been considered to control infectious diseases effectively [34,35,36]. Nanomedicine has recently been used to increase immune responses to antigens for efficient vaccination, targeted delivery and sustained release of medications, and rapid and reliable disease detection and diagnosis at low cost [37,38].

In this review, we will discuss the structure and health problems of *K. pneumoniae* and treatment options and diagnostic techniques for *K. pneumoniae*–induced infectious diseases [39].

## 2. Traditional Detection and Treatment Methods

Clinical criteria for the identification of hvKp strains and related infections are often problematic; because the clinical definition for hvKp strains, which includes the occurrence of community-acquired infection and tissue invasion in a healthy host, prevents the detection of hvKp infection in immunocompromised patients or individuals that stay in a health care center, it is difficult to identify strains that lack clinical information [13]. For this reason, detection of iucA, iroB, peg-344, and prmpA2 genes and string testing are done to identify hvKp strains [13]. Because the delay of appropriate treatment for *Klebsiella* spp., infections is associated with adverse prognosis and increased mortality, rapid diagnosis is essential to manage these infections [40]. Many molecular methods are used for the detection of antibiotic-resistant *K. pneumoniae*, such as multiplex PCR assay [41], DNA microarray [42], Real-time PCR assay [43], single-colony whole-genome sequencing [44], Raman spectroscopic analysis [45], loop-mediated isothermal amplification (LAMP) [46], matrix-assisted laser desorption ionization-time-of-flight mass spectrometry (MALDI-TOF MS) [47], and chromogenic media [48]. The gold standard for confirming the presence of KPC is the detection of hydrolysis of a carbapenem by spectrophotometry testing and detection of *bla_KPC_* by the PCR technique [49]. There are several phenotypic tests to identify carbapenem-resistant *K. pneumoniae* strains. These methods, which have also been approved by the Clinical & Laboratory Standards Institute (CLSI), are the CarbaNP test, mCIM, and eCIM; these have good sensitivity and specificity for detecting different carbapenemase enzymes [50]. Another phenotypic method is boronic acid-based compounds used as reversible inhibitors of class C β-lactamases [51]. Table 1 summarizes the conventional detection methods of *K. pneumoniae* and compares their properties.

Routine antibiotic treatments used for the resistant *Klebsiella* spp. show different percentages of effectiveness. In a study reviewing the reports, the significance of each of the antibiotics was stated as follows: polymyxin (14%), carbapenem (40%), tigecycline (71%), polymyxin combinations (73%), and aminoglycosides (75%) [49]. To overcome bacterial resistance and maximize bacterial killing, combination therapies are sometimes suggested, which include a combination of colistin, tigecycline, and meropenem, which can be effective against KPC-producing isolates and colistin-producing strains [57]. New β-lactamase inhibitors have been developed to resist hydrolysis by class A carbapenemases and ESBLs, including LK-157, NXL104, and BLI-489 [58]. Today, various methods are being developed to treat drug-resistant *K. pneumoniae* infections, including probiotics, phytochemicals, metal salts, and bacteriophage therapy [59]. One of the treatment methods considered an alternative to antibiotic treatments is bacteriophages. An animal model study has shown that bacteriophages provide reasonable protection against respiratory infections and other infections caused by *K. pneumoniae,* such as bacteremia and liver abscesses [60]. Probiotic refers to living microorganisms that benefit the host if present in sufficient quantities. Studies have shown that the administration of probiotics has been associated with a reduction in pneumonia incidence, a decrease in the duration of cold infection, and protection against respiratory pathogens [61]. Several studies have examined the effect of probiotic *Bifidobacterium longum* 51A and lactic acid bacteria (LAB) on *K. pneumoniae* [62]. Despite this, there are still many challenges in treating antibiotic-resistant *K. pneumoniae* infections, and research is ongoing to investigate other factors and treatment approaches [63,64].

## 3. Nanotechnology-Assisted Approaches for Effective Detection of *K. pneumoniae*

### 3.1. Nanoparticle-Assisted Multiple Cross-Displacement Amplification

Numerous isothermal amplifying approaches using nanostructures to amplify nucleic acids in water baths or a simple heating block have been widely reported. The multiple cross displacement amplification (MCDA) test, developed by Wang et al., is a quick and easy method for amplifying nucleic acids at a fixed temperature in under 40 min [65]. For example, the detection of *K. pneumoniae* by label-free MCDA coupled with nanoparticle-based biosensors was developed by Wang et al. [66]. The MCDA reaction was carried out for only 30 min at a constant temperature (65 °C), and the amplification findings were directly presented utilizing a lateral flow biosensor (LFB). The results demonstrated that reaction products might be detected in clinical samples with as few as 100 fg and 4.8 CFU of pure *K. pneumoniae* templates and as few as 480 CFU in 1 mL of spiked clinical specimens. All *K. pneumoniae* strains tested were positive for label-free MCDA-LFB analysis, while all non–*K. pneumoniae* strains tested were negative for label-free MCDA-LFB analysis, showing the label-free MCDA-LFB assay’s excellent specificity. The label-free MCDA-LFB test was further enhanced with Antarctic heat-sensitive uracil-DNA-glycosylase (AUDG) to reduce carryover contamination and remove misleading data. As a result, the label-free MCDA-LFB assay combined with the AUDG enzyme proved to be a quick, easy, sensitive, and reliable method for detecting the target pathogen. It also could efficiently avoid carryover contamination, making it a valuable tool for clinical diagnosis, “on-site” identification, and primary quarantine [66].

A similar study reported that an MCDA assay for identifying *K. pneumoniae* could produce positive results after 40 min of isothermal amplification [67]. For the quick readouts of MCDA amplification, colorimetric indicators and an Au NP LFB were used. The LOD of this assay was 100 fg per reaction at 65 °C, which was validated by real-time turbidimeters to be the best amplification temperature. For the MCDA assay’s specificity, all 30 clinical-source *K. pneumoniae* strains were positive, whereas all non–*K. pneumoniae* strains from 31 different species were negative. The approach’s practicability was used to identify *K. pneumoniae* strains in sputum samples (24 CFU per reaction) and DNA templates from 100 sputum samples using the MCDA-LFB technique. The MCDA test correctly recognized all of the sputum samples that were positive for *K. pneumoniae* (30/100) utilizing the culture technique, and its identification sensitivity was greater than that of the polymerase chain reaction (PCR) (25/100). As a result, the MCDA test for *K. pneumoniae* and the gold nanoparticle LFB is a straightforward, specific, and accurate approach for detecting *K. pneumoniae* in clinical specimens [67].

In addition, in another study, using MCDA and Au NPs LFB, a simple approach for detecting *Pseudomonas aeruginosa* (*P. aeruginosa*) was developed [68]. This approach performed the reaction in 40 min at an optimal constant temperature (67 °C). An LFB could visually detect the reaction product, obviating the need for specialized equipment. The *P. aeruginosa*-MCDA-LFB technique was highly specialized for *P. aeruginosa* and accurately separated it from other infections. It was possible to identify only 10 fg of the genomic DNA template (from pure culture). The assay had the same specificity and sensitivity as the reference (culture-biochemical) approach for detecting *P. aeruginosa* in clinical sputum samples [68].

To discover the mcr^−1^ gene, Gong et al. employed a similar approach and developed an MCDA linked with the detection of amplified products using an Au NP LFB system [69]. The MCDA-LFB test was done at an isothermal temperature (63 °C) for only 30 min during the amplification phase. The reaction products were directly recognized using LFB, which produced results in less than 2 min. The whole test process took about 60 min, from template extraction to the results from evaluation. All of the 16 mcr-1-producing strains were positive, while all of the non-mcr-1 isolates yielded negative findings, demonstrating the analytical sensitivity of this approach. In pure culture, the specificity of the mcr-1-MCDA-LFB test was as low as 600 fg of plasmid DNA per response, while in spiked fecal samples, it was around 4.5 × 10^3^ CFU/mL [69].

**Figure 1:**
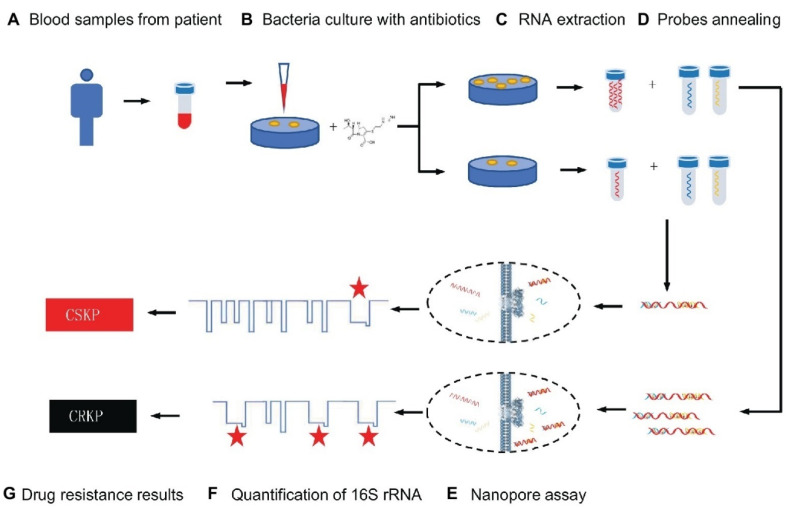
Schematic representation for carbapenem-resistant *K. pneumoniae* nanopore assay adapted from [70], Frontiers, 2019.

Because of its low cost, good effectiveness, and real-time identification, the nanopore test has recently been employed for screening biomarkers of diseases [70]. For example, Niu et al. separated CRKP from carbapenem-sensitive *K. pneumoniae* (CSKP) by detecting increased amounts of extracted 16S ribosomal RNA (16S rRNA) from bacterial culture using imipenem, showing that CRKP’s growth was unaffected by the antibiotic [70]. The quick and ultra-sensitive identification of 16S rRNA was enabled by specific signals from the single-channel recording of 16S rRNA bound with probes by MspA nanopores. This study demonstrated that only 4 h of CRKP growth time was required for the nanopore analysis to discriminate between the two proteins. The test takes about 5% of the time of the disk diffusion approach while achieving equivalent precision (See Figure 1) [70].

### 3.2. Nanoparticle-Assisted Loop-Mediated Isothermal Amplification

There have been numerous investigations on the LAMP (loop-mediated isothermal amplification) assay for pneumoniae identification. However, this diagnostic system often produces false positive results due to a high rate of non-specific reactions caused by the formation of hairpin structures, self-dimers, and mismatched hybridization [71]. Recent advances have proven that nanomaterials can effectively inhibit undesired amplification and reduce non-specific signals effectively [72]. For example, a LAMP-coupled nanoparticle-based LFB assay (LAMP-LFB) was developed for the specific detection of pneumoniae (P-LAMP-LFB) [73]. The optimal temperature for this experiment was proven to be 65 °C, and six primers corresponding to the P1 gene of pneumoniae were prepared. Within 2 min, LFB was able to evaluate the amplified outputs accurately. The P-LAMP-LFB test recognized DNA templates of pneumoniae exclusively, with no cross-reactivity with other infections. The LOD of this approach in pure cultures was about 600 fg of the DNA templates, which was in perfect agreement with agarose gel electrophoresis assay and colorimetric indicator identification. This technique was compared to real-time PCR analysis on 209 oropharyngeal swab specimens obtained from children suffering respiratory tract infections. Positive rates of pneumoniae were 47.8% and 31.6%, respectively, using the LAMP-LFB and real-time PCR assays. Compared to the real-time PCR approach, the LAMP-LFB assay exhibited greater selectivity [73].

### 3.3. Optical Nanosensors

**Figure 2:**
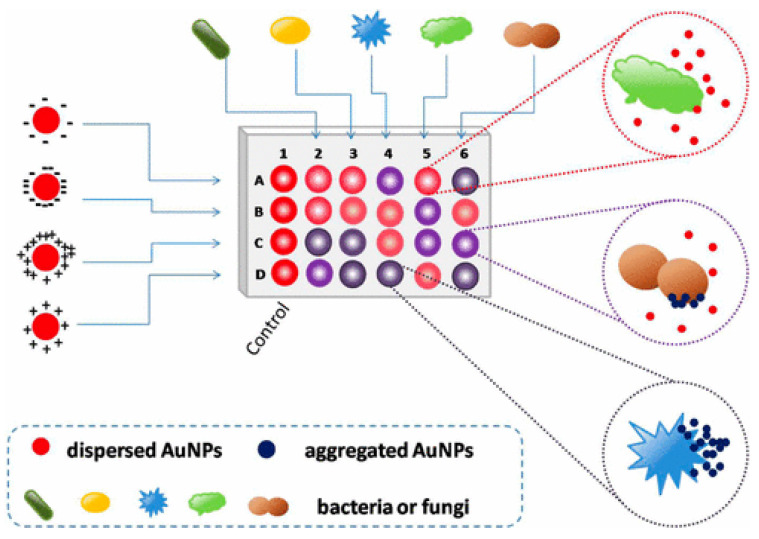
Schematic representation of the colorimetric nanosensor with four types of coated AuNPs. The interactions of microorganisms and AuNPs result in color changes. In the diagram, column 1 represents the blank control, and other columns show various organisms. Rows A to D represent four coated AuNPs, adapted from [80], ACS, 2017.

Colorimetric and fluorescence sensor arrays have generated great interest in recent years because of their capacity to differentiate a wide range of bacteria with a high recognition rate [74]. These biosensors rely on the optical characteristics of the sensor surface being altered by the bound analyte, and these changes are subsequently communicated to a detector [75,76,77]. The development of nanotechnology and the application of nanomaterials’ advantages on a biosensor platform allow for an improvement in the biosensors’ sensing parameters. Nanomaterials are employed to great advantage in nanosensors as a label-free detection approach because of their capacity to induce SPR [78,79]. In this light, a colorimetric nanosensor was developed for selectively identifying bacteria like *K. pneumoniae*. Four gold nanoparticles (AuNPs) with different surface charges were employed as sensing components. The interactions of AuNPs with microbes resulted in visible hue shifts that could be seen with bare eyes. A total of 15 bacteria exhibited distinct reactions that were effectively distinguished using linear discriminant analysis (LDA). Microorganism combinations may also be determined with ease. This approach is simple, quick (less than 5 s), efficient, and visual, indicating that it could be used for pathogen detection and environmental sensing (See Figure 2) [80].

Surface plasmon resonance (SPR), a label-free optical biosensor, has been used to detect a variety of compounds [81]. Additionally, surface-enhanced Raman scattering (SERS) boosts a Raman spectrum manifold’s amplitude and has been used in conjunction with other techniques to find bacterial cells in blood medium [81,82]. The nanophotonic interferometric biosensor is another cutting-edge biosensing device that offers a quick way to identify nosocomial pathogens for the diagnosis of illnesses [83]. For example, a recent study established a label-free approach for quickly detecting clinically relevant multilocus sequencing typing (MLST)-verified quinolone-resistant *K. pneumoniae* strains. This approach was also used to recognize three quinolone-resistant *K. pneumoniae* strains from colony samples, ST15, ST11, and ATCC70063 (control), which are the most common quinolone-resistant *K. pneumoniae* strains in East Asia. A multivariate statistical method combined with a drop-coating deposition surface-enhanced Raman scattering (DCD-SERS) approach was used to identify the colonies. The process had a correlation coefficient of 0.98 LOD of 100 pM rhodamine 6G, strong repeatability (relative standard deviation of 7.4%), and a Raman enhancement factor of 11.3 × 10^6^. Compared to *Escherichia coli* (*E. coli*), all quinolone-resistant *K. pneumoniae* strains displayed similar spectral Raman shifts (high correlations) and distinct Raman vibrational modes. The suggested DCD-SERS strategy, in combination with a multivariate statistics-based identification method, performed exceptionally well in subtyping *K. pneumoniae* strains and distinguishing identical microorganisms. As a result, the label-free DCD-SERS approach combined with the computational decision-supporting method could be a valuable technique for accurately identifying clinically relevant *K. pneumoniae* strains [84].

### 3.4. Cantilevers-Based Nanosensors

**Figure 3:**
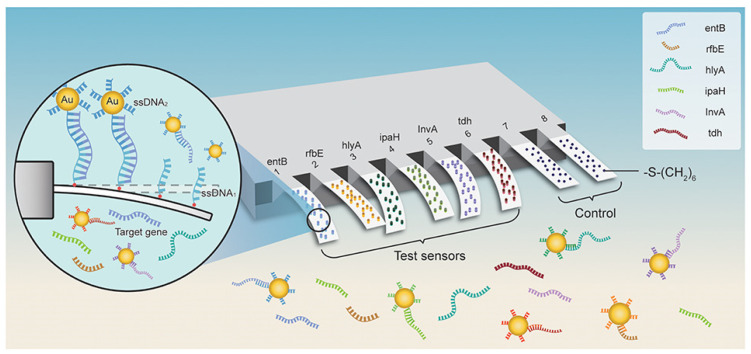
Schematic representation for microcantilever array biosensor modified with gold nanoparticles for foodborne bacteria detection, adapted from [86], Frontiers, 2019.

As a result of their high responsivity and ease of integration, cantilevers are gaining increasing popularity as new-generation biosensors. Microcantilever-based nanosensors are highly appealing for biomedical purposes because of their fast and real-time analysis, ultrasensitive features, and label-free potential [85]. For example, a new array biosensor made up of a microcantilever and gold nanoparticle could detect ultralow quantities of pathogenic bacteria, such as *Listeria monocytogenes, Shigella* spp., *Vibrio parahaemolyticus*, *Staphylococcus aureus*, *E. coli O157:H7*, *Salmonella* spp., *Listeria monocytogenes*, *Shigella* spp., and many others [86]. The process was significantly quicker than traditional methods that need PCR amplification or germi-culturing. Six pairs of ssDNA probes (ssDNA1 + ssDNA2) were designed and verified based on sequence examination of the bacteria’s particular gene. The -S-(CH2)6 parts were attached at the 5′ end of ssDNA1 probes and immobilized as a self-assembled monolayer (SAM) on the surface of cantilevers and finally integrated with Au NPs. In addition, the 6-mercapto-1-hexanol SAM were attached to reference cantilevers to remove interferences from non-specific interactions (Figure 3). Microcantilever array sensors and Au NP platforms can detect quickly and precisely various bacteria with working ranges of 3 to 4 orders of magnitude and LOD of 1–9 cells/mL. There was negligible cross-reaction between the probing of various organisms. This has a promise for quick, combinatorial, and extremely sensitive identification for environmental, clinical, and food items [86].

### 3.5. Electrochemical Nanosensors

Electrochemical nanosensors have potential as a diagnostic tool for people and animals. These sensors can detect pathogens and biomarkers in bodily fluids such as urine and blood [87]. For example, the development of a label-free DNA biosensor for the identification of *K. pneumoniae* that can also diagnose other types of bacterial infections was reported by Zhang et al. [88]. In this light, on a glassy carbon electrode, graphene oxide (GO) and indole-5-carboxylic acid (ICA) were successfully deposited, and the resultant ICA/GO (rGO) hybrid film was used to immobilize oligonucleotides on an ssDNA sequence (single-stranded DNA). In optimized conditions, the electrochemical nanosensor showed outstanding performance. A considerable change was seen after hybridizing the target probe with ssDNA under optimal conditions. Differential pulse voltammetry was used to investigate hybridization with three-base mismatched, one-base mismatched, noncomplementary, and complementary DNA targets. With a linear range of 1 × 10^−6^ to 1 × 10^−10^ M, the suggested method could identify target DNA as low as 3 × 10^−11^ M, demonstrating great sensitivity of the nanosensor. The nanosensor showed fast analysis time, devoid of indicators, and had a high degree of specificity. As a result, the proposed nanoplatform can help diagnose *K. pneumoniae* and other pathogen-related infections [88].

Another work presented a glassy carbon electrode (GCE) enhanced with graphene and Au-NPs as a new electrochemical nanosensor for DNA identification. After that, electrochemical impedance spectroscopy, cyclic voltammetry (CV), and scanning electron microscopy (SEM) was used to analyze Au-NPs/Gr/GCE (EIS). In addition, differential pulse voltammetry (DPV) using methylene blue (MB) as the hybridization marker was performed to identify hybridization processes. The sensor’s LOD and dynamic range for the target DNA sequences were 2 × 10^−13^ mol/L and 1 × 10^−12^ to 1 × 10^−7^ mol/L, respectively. In the presence of mismatched and non-complementary DNA sequences, the DNA nanosensor demonstrated remarkable selectivity for recognizing complementary DNA sequences. The findings showed that the Au-NP/Gr nanostructure is a potential substrate for developing high-performance catalytic systems for KPC measurement [89].

### 3.6. Biomimetic Nanosensors

Nature is a source of inspiration for solving biological problems. Potentiometric, voltammetric, and impedance spectrum sensors are among the biomimetic nanosensors that mimic the behavior and operation of living organisms by being modified with nanomaterials and specially designed biomimetic materials [90]. A new biosensor was designed using graphene and two-photon polymerization to provide an improved biosensor for detecting motile bacteria. Around graphene-based sensing electronics, a cage with a directed micro-architecture was covered that was inspired by venous valves. The designed 3D-mucro architecture enables motile cells to move from the outside of the cage to the center area, leading to an accumulation of bacteria around the core sensing zone, which improves the received signal. The concentrating effect has been shown in cell cultures, ranging from 10^1^ to 10^9^ CFU/mL. Fluorescence evaluation indicated a signal enhancement of 3.38–3.5 fold. Identifying cellar metabolites improves the pH sensor by 2.14–3.08 fold. Electrical tests showed an increase in current of 8.8–26.7 fold. The suggested architecture enables the construction of smart biomedical sensors using bio-inspired 2D materials and 3D printing in a novel manner [91]. Table 2 summarizes the nano-based detection method of *K. pneumoniae*.

## 4. The Application of Nanomaterials for Treatment of *Klebsiella pneumoniae*

Antimicrobial resistance (AMR) threatens human well-being globally. The mortality rate due to AMR is expanding and is anticipated to reach 50 million by 2050 [96]. Microorganisms such as *Klebsiella* spp. have increased resistance to accessible antimicrobial specialists. Currently, there is a pressing need for alternative medicine to address the AMR risk [97]. One of these is nanotechnology-based pharmaceuticals [98]. Nanoscience allows us to synthesize a variety of nanoparticles (NPs) from bulk materials [99,100]. Utilizing nanoparticles to transport drugs, heat, light, or other chemicals to particular types of cells is currently under development [101,102]. The use of nanomaterial-based therapeutics, which have the ability to circumvent current defenses linked to acquired drug resistance, is a potential strategy for treating bacterial infections that are challenging to cure. Additionally, nanomaterials’ distinct dimensions and physical characteristics enable them to target biofilms and treat resistant illnesses [103]. Ag nanoparticles (AgNPs) have a large surface area and small size, and they are one of the most effective antimicrobials [104]. This encourages contact between them and their targets, such as bacterial cells [105]. Tsung-Ying Yang et al. fabricated antibacterial silver nanoparticles (Ag NPs) that were restricted to a mesostructured material and used as an Ag/80S bioactive nanocomposite against *K. pneumoniae*. Ag/80S had a 7.5 nm mesoporous size and 307.6 m/g surface area, according to textural analysis. The Ag/80S UV–Vis spectrum and TEM pictures exhibited a homogeneous Ag NP size distribution with a mean size of ~3.5 nm. The minimum inhibitory concentration (MIC) for KP isolates was 0.25 to 0.5% (2.5 to 5.0 mg/mL). The inspired Ag/80S adhered to and distorted bacterial cells, causing a time-dependent amassing of reactive oxygen species (ROS), which resulted in bacterial death, according to the results of the mechanistic investigation [106]. In a separate investigation, Aghigh Dolatabadi and colleagues examined the inhibitory effects of commercial and green Ag NPs on OxqAB efflux pump genes in ciprofloxacin-resistant *K. pneumoniae* strains. The effectiveness of the synthetic nanoparticles was determined by comparing the activity of the antiefflux pump with that of commercial Ag NPs. The *oxqAB* gene expression levels were lowered in ciprofloxacin-resistant isolates in the sub-minimum inhibitory concentration (MIC) of both Ag NPs, suggesting that efflux pumps could be a promising target for biosynthesized AgNPs. The expression of the *oxqAB* gene was lowered in both Ag NPs’ subMIC, although biosynthesized Ag NPs showed better bactericidal effects than commercial Ag NPs [107]. In addition, Sanjay Chhibber et al. investigated the therapeutic efficiency of histidine-capped silver nanoparticles delivered through microemulsions in the treatment of *K. pneumoniae*–induced infection. Thixotropy, texturing, differential scanning calorimetry, and release kinetics were used to construct and analyze the emulgel. The treatment’s effectiveness was assessed using bacterial load, histology, tissue repair, and other infectious indicators. When compared to untreated animals, the developed emulgel showed a remarkable in vivo antimicrobial effect of the histidine-capped silver nanoparticle preparations when applied topically, associated with decreased microbial infection, tissue regeneration, and improved skin healing, as well as reduced systemic inflammation like malondialdehyde, myeloperoxidase, and reactive oxygen and nitrogen intermediates [108].

Researchers in earlier trials combined two therapeutic approaches to treat bacterium-induced infectious disease. For example, Mai I. El-kaliuoby and colleagues investigated the synergistic effects of combining exposure to a low frequency pulsed magnetic field (LF-PMF) with the administration of AgNPs. KP bacterium, which is Gram-negative, was tested under varied AgNP concentrations and contacted LF-PMF at various frequencies. The optimal synergistic impact is obtained by achieving the highest inhibitory concentration of AgNPs and the lowest ELF-PMF resonance frequency that inhibits bacterial growth the most. Exposure to 20 Hz PMF for 30 min with a supplement of 150 ppm Ag NPs resulted in a highly synergistic impact, with a 90 percent increase in growth inhibition, according to growth kinetics [109]. In a separate study, Kate Kotlhao and colleagues used various chemical procedures to produce three different types of nanoparticles, silver (Ag), zinc oxide (ZnO), and titanium dioxide (TiO_2_), and compared their antibacterial efficacy. The nanoparticles were in the nanoscale range, according to TEM (1100 nm). The various absorption bands of the fabricated nanoparticles were revealed using FTIR and UV-Vis spectroscopy, respectively. Ag NPs displayed an antibacterial effect more than TiO_2_ and ZnO NPs against a variety of bacteria. *K. pneumoniae* showed the greatest inhibition. The findings shown that the antibacterial effect of NPs increases as the concentration of NPs increases [110].

**Figure 4:**
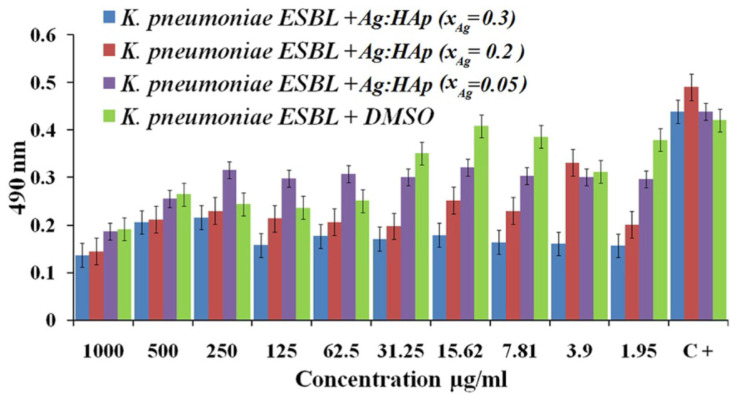
Antibacterial effect of Ag:Hap-NPs (xAg = 0.3, 0.2, and 0.05) on KP, adapted from [111], Springer, 2012.

Ciobanu et al. fabricated Ag-doped crystalline hydroxyapatite NPs (Ag:HAp-NPs) (Ca-xAgx(PO_4_)_6_(OH)_2_, xAg = 0.3, 0.2, and 0.05) via a coprecipitation method and investigated their antibacterial capabilities. The antibacterial effect of Ag:HAp NPs against *Citrobacter freundii*, *Providencia stuartii*, *S. aureus*, *K. pneumoniae*, and *Serratia marcescens* was studied. The results revealed that *S. aureus*, *K. pneumoniae*, *P. stuartii*, and *C. freundii* had the highest antimicrobial effect, regardless of sample type. This illustrated the antibacterial activity of different values of xAg on *K. pneumoniae*. The antibacterial effect of samples with xAg = 0.2 and 0.3 was unaffected by the Ag:Hap NPs concentration, whereas samples with xAg = 0.05 showed little antibacterial effect up to 31.25 g/mL Ag:Hap NPs concentration (Figure 4) [111].

**Figure 5:**
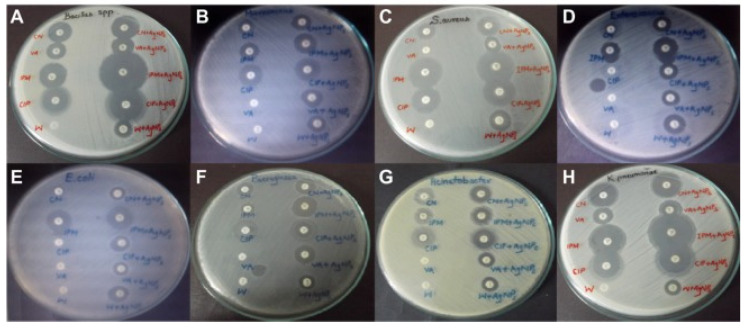
Zones of inhibition of multidrug-resistant bacteria by conventional antibiotics and AgNPs. (**A**) *Bacillus*; (**B**) *Micrococcus luteus*; (**C**) *Staphylococcus aureus*; (**D**) *Enterococcus faecalis*; (**E**) *E. coli*; (**F**) *Pseudomonas aeruginosa*; (**G**) *Acinetobacter baumannii*; (**H**) *K. pneumoniae*. Adapted from [112], Dove Medical Press Ltd., Macclesfield, United Kingdom, 2013.

Syed Zeeshan Haider Naqvi and colleagues studied the synergistic antibacterial effect of five conventional antibiotics and Ag NPs in order to determine an effective therapy for *K. pneumoniae* using the Kirby–Bauer disk-diffusion technique. They tested eight distinct multidrug-resistant bacterial species (*K. pneumoniae* is among them). According to the findings, the antibiotics and nanoparticles worked together to improve the antibacterial effect by 2.8-fold, demonstrating that NPs can be employed in conjunction with antibiotics to enhance their effectiveness against numerous pathogenic germs (Figure 5) [112].

Au NPs are an antibacterial nanoparticle that has been used as a treatment agent for *K. pneumoniae* in recent decades. Ayaz Ahmed and colleagues tested the antibiofilm efficiency of gold nanoparticles linked with chlorhexidine (Au CHX) towards *K. pneumoniae*. MTT assay was used to determine biofilm inhibition and eradication, and florescence and AFM microscopy were used to confirm these findings. A surface plasmon resonance (SPR) band maxima at 535 nm, spherical shape, and polydispersity with sizes ranging from 20 to 100 nm were observed for Au-CHX nanoparticles. The biofilm construction and metabolic effect inside biofilms of KP reference and three tested clinical isolates were fully suppressed at micro molar concentrations (i.e., 25 and 100 M) of Au-CHX, respectively. The concentrations of 70 and 100 µM eradicated the *K. pneumoniae* established biofilms [113].

Antimicrobial substances such as ZnO and ZnO NPs are becoming more widely used as antibacterial agents. Jehad M. Yousef et al. investigated the minimum inhibitory concentration (MIC) or minimum bactericidal concentration (MBC) of ZnO NPs against a variety of pathogens, including *K. pneumoniae*. According to the findings, nano-Zno has an excellent bacteriostatic impact but a weak bactericidal activity against the whole pathogens studied. ZnO NPs could be a viable antibacterial agent due to the inexpensive cost of manufacture and great antimicrobial effectiveness and could be used in a variety of industries to address safety concerns [114].

Doping of some ions in the ZnO NPs can improve their antibacterial activity. Accordingly, Hameed et al. used the coprecipitation approach to fabricate pure ZnO and Nd-ZnO NPs with nanorod and nanoflower shapes, respectively. Antibacterial experiments on *E. coli* and *K. pneumoniae* ESBLs revealed that the Nd-ZnO NPs had a higher antibacterial activity than the pure ZnO NPs [115].

Antibacterial properties of titanium oxide (TiO_2_) NPs have been investigated against *K. pneumoniae*. Many studies have used TiO_2_ nanoparticles. Venkatasubbu et al. reported TiO_2_ and ZnO’s ability to preserve food. A wet chemical process was used to synthesis TiO_2_ and ZnO NPs. Both materials’ antibacterial activity was examined to ensure that they were effective as food preservatives toward *Salmonella typhi*, *Shigella flexneri*, and *K. pneumoniae*. The findings shows that ZnO and TiO_2_ NPs hinder growth of *Salmonella*, *Klebsiella* and *Shigella* spp. [116]. B.K. Thakur and colleagues published a paper in which they described a method for synthesizing TiO_2_ NPs using Azadirachta indica leaves. *E. coli*, *B. subtilis*, *S. typhi*, and *K. pneumoniae* were used to study the antibacterial efficacy of the produced ZnO and TiO_2_ NPs compound. The findings of this investigation show that TiO_2_ NPs prevented the full growth of the examined bacteria. The lowermost MBC value, i.e., 83.3 μg/mL was detected toward *K. pneumoniae* [117]. One-dimensional CuO/TiO_2_ nanofibers with excellent antibacterial effect were synthesized and studied by Ayman Yousef and colleagues. The antibacterial effect was determined by calculating the MIC against *K. pneumoniae* as a model organism, and the manufactured NPs exhibited high antibacterial activity against *K. pneumoniae* based on MIC measurements compared to when they were utilized separately [118].

Cadmium nanoparticles are among the most promising and new treatment agents for infectious diseases. Ashwani Kumar and coworkers tested the antibacterial susceptibility of CdS NPs (pure and 1% Cu doped) toward bacteria *S. aureus*, *Salmonella typhimurium*, *P. aeruginosa*, *E. coli*, and *K. pneumoniae*. Using the well diffusion technique, the antimicrobial effect of the synthesized NPs was investigated by measuring the zone of inhibition in the concentration range of 1 mg/mL to 100 mg/mL of CdS NPs. The MBC and MIC were determined after the initial antibacterial qualitative test. Cu-doped CdS NPs were more effective than pure CdS nanoparticles, with MICs ranging from 0.078 to 0.52 mg/mL, as compared to pure CdS NPs, which had MICs ranging from 0.15 to 0.83 mg/mL [119].

In recent years, silica (SiO_2_) NPs have shown antibacterial properties. Na Xu and colleagues synthesized and then characterized SiO_2_ NPs by TEM and DLS techniques. They then tested their antibacterial effect toward *K. pneumoniae* using the minimum inhibitory concentration (MIC) procedure. The findings exhibited the influence of NP size on antibacterial ability; when compared to Sulfamethoxazole as control, the produced NPs with a size of 40 nm demonstrated a good inhibitory efficacy against *K. pneumoniae* [120].

Rahman et al. described the influence of flexible and multimodal hybrid membranes (BC-SiO_2_-TiO_2_/Ag) that contain bacterial cellulose. This nanoplatform had Ag and TiO_2_ that awarded antibacterial properties, UV guarding, and photocatalytic and self-cleaning characteristics. The BC-SiO_2_-TiO_2_/Ag membranes showed good photoactivity and significant antibacterial efficacy towards five different bacterial strains [121].

Bushra Al Edhari and colleagues studied the synergistic effect of Ag NPs, Ni NPs, and Al_2_O_3_ NPs against all *K. pneumoniae* strains separately and in combination. Ag/Ni NPs and Ag/Al_2_O_3_ NPs exhibited much more antibacterial and antibiofilm activity against *K. pneumoniae* [122]. Table 3 gives a set of nanostructures that have been used to treat *K. pneumoniae*–related infections.

The problem of hospital-acquired infection is now compounded by the rise of antimicrobial resistance to antibiotics. Hospitals are loci of the intensive deployment of antibiotics. This, in turn, imposes intense selection pressure on the microorganisms, which generally have the capacity to deploy or evolve resistance mechanisms. One of practical techniques to combat hospital-acquired infections is photocatalytic nanomaterials. Photocatalytic antimicrobial coatings, especially those based on titanium dioxide, which is the most extensively investigated material, appear to be capable of keeping the environmental microbial burden of hospital surfaces close to zero [123,124]. Reid et al. sprayed titanium dioxide–based photo-catalytic coating onto six surfaces in a four-bed bay in a ward and compared them under normal lighting to the same surfaces in an untreated ward in order to assess the impact of a photocatalytic antimicrobial coating at close-to-patient, high-touch sites. These surfaces included right and left bed rails, bed controls, bedside lockers, overbed tables, and bed footboards. Significantly lower levels of bacteria were present on treated surfaces than on control sites, and this difference between treated and untreated surfaces grew over the course of the investigation [124].

**Table 3:** Nanostructures that have been used to treat *K. pneumoniae*–related infectious.

Substrates	NP Size (nm)	Key Features	Ref.
Ag-rifampicin	15–18	Greater antimicrobial effect than free drug	[125]
Ag	29–50	High antibacterial effect	[126]
Au-imipenem	12–27	Strong synergistic antibacterial effect	[127]
Ag	34–90	Low minimal inhibitory concentration	[128,129]
Ag	3	100% bactericidal effect at 0.05 g/mL	[130]
TiO_2_ and Ag	20 and 90	Antibiotics and nanoparticles are combined; they have a synergistic impact	[131]
TiO_2_	-	High antibacterial activity	[132]
TiO_2_	50 and 100	Good antibacterial nature	[133]
TiO_2_ + L. lactis	-	Reliable and operative inorganic antimicrobial agents	[134]
CML@Ag-NPs and CML@Au-NPs	40–60	Obtained MIC values for Ag and Au NPs were 0.5 and 370 ppm	[135]
Ag/AgCl-imipenem (IPM)	55-89	Synergetic effect between the IPM antibiotic and Ag/AgCl NPs	[136]
ZnO	6–18	The antibacterial effectiveness of ZnO NPs-E was 40 g/mL, which was higher than previously reported values	[137]
ZnO	94	Low MIC and MBC in comparison with those obtained for imipenem and meropenem antibiotics	[138]
ZnO	11–25	During a lunar eclipse, ZnO NPs have a better antimicrobial activity than on a regular day	[139]
Cy-da/ZnO	29–35	The highest antibacterial activity for Cy-da/ ZnO NPs in comparison with ZnO NPs	[140]
ZnO	45–50	Green synthesis with proper size	[141]
ZnO	88	At dosages of 0.50 to 0.75 mM, the NPs-treated *K. pneumoniae* was five times less infectious	[142]
ZnO/bovine serum albumin(BSA)	11	BSA improved antibacterial effect of ZnO NPs	[143]
ZnO/Zeolite	-	In sublethal levels, it has strong antibiofilm efficacy against *K. pneumoniae*	[144]
Ag/ZnO	143 and 154	The toxicity of nanoparticles strongly depend on surface charge effect	[145]
ZnO	90–110	Eco-friendly and simple method for synthesis of ZnO NPs	[146]
Fe/Co-ZnO	-	6% Fe and 4% doped ZnO show the maximum antibacterial effect	[147]
Carbon cloth/Ag/ZnO	-	Cloth carbons improved antibacterial activity of metal or metal oxide NPs	[148]
Ga-ZnO	-	The bioactivity of undoped and Ga-ZnO nanocrystals is significantly improved	[149]
Ag@SiO_2_	118	Antibacterial activity greater than than Ag and SiO_2_ NPs separately	[150]
Mn-TiO_2_	150	100% reduction of *Klebsiella pneumoniae* within 120 min under sunlight	[151]
Ag-TiO_2_	163	Superior antimicrobial activities to Ag and TiO_2_	[152]
N-TiO_2_, C-TiO_2_, N-T-TiO_2_, and Pd-C-TiO_2_	5–60	Under visible-light irradiation, Pd-C-TiO_2_ had the maximum capacity for bacterial inactivation	[153]
Au-Ag	12–67	The synthesis procedure is environmentally benign because it does not use any solvents or harmful chemicals	[154]
Au	5.5–10	Remarkable bactericidal activities against polymyxin-resistant *Klebsiella pneumoniae*, which are superior to clinical antibiotics	[155]
Fe_3_O_4_	24	Good additional antimicrobial	[156]

## 5. Conclusions, Challenges and Future Perspective

*K. pneumoniae* has been described as an immediate threat to human health. The grade of pathogenicity and virulence of *K. pneumoniae* and the emergence of new MDR strains has led scientists to seek new antibacterial compounds. Alternative methods include the use of multiple antibiotics, phage therapy, probiotics, and phytochemicals; however, each has its limitations. The delivery of antibacterial agents, the clinical acceptance of several nanotechnology-based devices for the diagnosis of pathogen infection, and the advancements in medical equipment with antimicrobial coatings have highlighted the promising effect of nanoscience on microbial infectious diseases. Nanostructures with specific physicochemical characteristics have allowed for great specificity and sensitivity in the detection of *K. pneumoniae*, as well as quick readout. Furthermore, covering medical equipment with antimicrobial nanoparticles, particularly Ag, has significantly decreased device-related bacterial infection and biofilm growth, as well as improved wound repair.

While nanomedicine is promoting a range of developments in the antimicrobial area, practical design of antimicrobial nanoscience still faces significant obstacles. The fast appearance of novel nanomaterials that have been clinically disproved will need a more thorough evaluation of their long-term safety and biocompatibility. The design and marketing of sophisticated nanostructured materials (e.g., targeted multimodal nanostructures) is hampered by the inability to mass produce them with low batch-to-batch variance. Novel monitoring approaches are also critical for fast in vitro and in vivo testing of nanoparticles and their biophysicochemical properties, such as composition, size, structure, zeta potential, targeting ligand, and surface area. Additionally, other properties such as loading performance, stability, matrices, release kinetics, immune response, pharmacokinetics, and biodistribution can be different for each approach. Moreover, designing more clinically useful animal studies, knowing the microenvironment of bacterial infection sites, recognizing pathways of new biomarkers and microbial pathogenesis, and lowering burdensome regulations can all help with the successful translation of antimicrobial nanodevices. We should anticipate many more nanotechnologies to be introduced into the clinic to address every element of microbial illness as antimicrobial nanomedicine advances.

Despite these remarkable advances, nanotechnology’s full promise in the management of microbial illness, notably in the fields of antimicrobial treatment and vaccinations, is still far from being exploited. Some suggestions for improving the efficiency of nanoparticles to address *K. pneumoniae* are (i) taking full advantage of the EPR effect in infection sites; (ii) development of theranostic nanoparticles by combining diagnostic and therapeutic agents; and (iii) the use of nanotechnology along with gene silencing technologies such as the antisense strategy and RNA interference (RNAi).

## Figures and Tables

**Table 1:** Conventional detection methods of *K. pneumoniae*.

Detection Method	Principle	Advantage	Disadvantage	Ref.
**Multiplex polymerase chain reaction**	Multiple targets can be detected simultaneously in a single reaction using separate primers for each target and using two or more probes.	- More information with fewer samples - Higher throughput - Cost-effective - Time-saving - Less input material - Increased accuracy of data normalization - Fewer pipetting errors	- Self-inhibition among different sets of primers - Low amplification efficiency - No identical efficiency for different templates	[52]
**DNA microarray**	A group of microscopic DNA patches is used to genotype different parts of a genome or to evaluate the expression levels of several genes at once linked to a solid surface.	- Low cost - Signal amplification is not needed - Independent of genome sequence - Elimination of artifacts from spotting	- Hybridization is dependent upon the length of sequences spotted - Labeling efficiency of dyes is an issue - Little information is generated for genes without expression in the reference/control sample - Handling of clones	[53]
**Single-colony whole-genome sequencing**	The process determines the DNA sequence of an entire genome and a brute-force approach to problem-solving uses a genetic basis at the core of a disease.	- Flexibility - The entire genome is scrutinized - Diagnosis of genetic mutations - Identification of genetic carriers of recessive diseases - Identification of genetic drivers of tumors and new biological therapies	- Less efficient at predicting some conditions than biochemical tests - Risk of false positives for mutations - Expensive - Privacy of data - Large capacity and cost required for storing data	[44]
**Raman spectroscopic analysis**	This uses light to stimulate (produce) molecular vibration in a sample, then interprets this interaction to perform a chemical analysis. It is based on the inelastic scattering of light that takes place when light interacts with matter.	- Label-free bacterial detection, identification, and antibiotic susceptibility testing in a single step - Interrogation of individual bacterial cells - Characterizes different bacterial phenotypes - Non-invasive, and non-destructive —tool for identifying microbes - Identifies microbes directly from human body fluids - Easy sample preparation	- Weak Raman signal from bacterial cells - Numerous bacterial species and phenotypes - No commercial databases of microbial Raman fingerprints - High input costs, complicated instrumentation, and specialized operators	[54]
**Loop-mediated isothermal amplification (LAMP)**	Amplifies DNA under isothermal conditions by using a DNA polymerase with high displacement strand activity and a set of specifically designed primers to amplify targeted DNA strands.	- Extremely high specificity - Conducted without the demand for expensive thermocyclers - Simple and easy selection of genes - No background interference - Ease of use and low-cost setup	- Detects false positives - Indirect detection of amplification products - Nonspecific amplification - Absence of temperature gating mechanisms	[55]
**Chromogenic media**	Inclusion of chromogenic enzyme substrates targeting microbial enzymes	- High detection rate for target pathogens - High differentiation of mixed cultures - Faster results (compared to the traditional method) - Reliable visual detection - Additional testing possible directly from the media	- Expensive media containing chromogenic substrates	[56]

**Table 2:** Nano-based detection method of *K. pneumoniae*.

Detection Method	Principle	Advantage	Disadvantage	Ref.
**Nanoparticle-assisted multiple cross-displacement amplification**	Amplifies the circular DNA template with the use of random primers and DNA polymerase. Within a few hours, the DNA may be amplified over 10,000 times.	- low cost - rapid isothermal amplification - more specificity and sensitivity than LAMP or MACD - visual inspection of color changes - point-of-care testing	- fails to amplify sequences with secondary structures - false positives	[92]
**Optical nanosensors**	Utilizes the altered optical characteristics of the nanometric surface of the sensor brought about by the bound analyte, and these altered optical characteristics are then sent to a detector.	- allows quantitative measurements in the intracellular environment - high detection limit	- expensive equipment	[93]
**Cantilever-based nanosensors**	A biomolecular interaction produces a change in the mechanical behavior of the transducer (a movement at nanometer scale), which can be measured and analyzed in real time.	- real-time and in situ measurement - detects molecular interactions without any kind of label - low cost of fabrication and mass production of extremely sensitive devices - can be operated in vacuum, gasses, and liquids	- mechanical–thermal noise - low detection limit - complex relation between the measured signal and the factors producing it	[94]
**Electrochemical nanosensors**	Transforms the interaction of an analyte with a receptor on the surface of an electrode into a useful analytical signal.	- requires low volume of sample - direct conversion of a biological event to an electronic signal - easy miniaturization - able to be used in turbid biofluids with optically absorbing and fluorescing compounds	- limited or confined temperature range - short or limited shelf life - cross-affectability of different gases - possible electrochemical interference	[95]

## Data Availability

Not applicable.
